# Machine learning is an effective method to predict the 90-day prognosis of patients with transient ischemic attack and minor stroke

**DOI:** 10.1186/s12874-022-01672-z

**Published:** 2022-07-16

**Authors:** Si-Ding Chen, Jia You, Xiao-Meng Yang, Hong-Qiu Gu, Xin-Ying Huang, Huan Liu, Jian-Feng Feng, Yong Jiang, Yong-jun Wang

**Affiliations:** 1 Department of Neurology, Beijing Tiantan Hospital, Capital Medical University, No.119 South 4th Ring West Road, Fengtai District, Beijing, 100070 China; 2 China National Clinical Research Center for Neurological Diseases, No.119 South 4th Ring West Road, Fengtai District, Beijing, 100070 China; 3 Institute of Science and Technology for Brain-Inspired Intelligence, Fudan University, Shanghai, 200433 China; 4 Beijing Advanced Innovation Center for Big Data-Based Precision Medicine (Beihang University & Capital Medical University), Beijing, 100091 China; 5 Advanced Innovation Center for Human Brain Protection, Capital Medical University, Beijing, China; 6 Clinical Center for Precision Medicine in Stroke, Capital Medical University, Beijing, China; 7 Research Unit of Artificial Intelligence in Cerebrovascular Disease, Chinese Academy of Medical Sciences, 2019RU018, Beijing, China; 8 Center for Excellence in Brain Science and Intelligence Technology, Chinese Academy of Sciences, Shanghai, China; 9 Chinese Institute for Brain Research, Beijing, China

**Keywords:** Machine learning, 90-day poor prognosis, TIA and minor stroke, Prediction models

## Abstract

**Objective:**

We aimed to investigate factors related to the 90-day poor prognosis (mRS≥3) in patients with transient ischemic attack (TIA) or minor stroke, construct 90-day poor prognosis prediction models for patients with TIA or minor stroke, and compare the predictive performance of machine learning models and Logistic model.

**Method:**

We selected TIA and minor stroke patients from a prospective registry study (CNSR-III). Demographic characteristics,smoking history, drinking history(≥20g/day), physiological data, medical history,secondary prevention treatment, in-hospital evaluation and education,laboratory data, neurological severity, mRS score and TOAST classification of patients were assessed. Univariate and multivariate logistic regression analyses were performed in the training set to identify predictors associated with poor outcome (mRS≥3). The predictors were used to establish machine learning models and the traditional Logistic model, which were randomly divided into the training set and test set according to the ratio of 70:30. The training set was used to construct the prediction model, and the test set was used to evaluate the effect of the model. The evaluation indicators of the model included the area under the curve (AUC) of the discrimination index and the Brier score (or calibration plot) of the calibration index.

**Result:**

A total of 10967 patients with TIA and minor stroke were enrolled in this study, with an average age of 61.77 ± 11.18 years, and women accounted for 30.68%. Factors associated with the poor prognosis in TIA and minor stroke patients included sex, age, stroke history, heart rate, D-dimer, creatinine, TOAST classification, admission mRS, discharge mRS, and discharge NIHSS score. All models, both those constructed by Logistic regression and those by machine learning, performed well in predicting the 90-day poor prognosis (AUC >0.800). The best performing AUC in the test set was the Catboost model (AUC=0.839), followed by the XGBoost, GBDT, random forest and Adaboost model (AUCs equal to 0.838, 0, 835, 0.832, 0.823, respectively). The performance of Catboost and XGBoost in predicting poor prognosis at 90-day was better than the Logistic model, and the difference was statistically significant(*P*<0.05). All models, both those constructed by Logistic regression and those by machine learning had good calibration.

**Conclusion:**

Machine learning algorithms were not inferior to the Logistic regression model in predicting the poor prognosis of patients with TIA and minor stroke at 90-day. Among them, the Catboost model had the best predictive performance. All models provided good discrimination.

**Supplementary Information:**

The online version contains supplementary material available at 10.1186/s12874-022-01672-z.

## Introduction

Stroke was the second leading cause of death worldwide and the leading cause of mortality and disability in China [1, 2]. In previous studies in multiple countries, stroke recurrence rate 90 days after TIA/minor stroke was 18%-20% [3]. About 40% of stroke survivors have a poor prognosis (modified Rankin Scale [mRS] score ≥3) between 1 month and five years after stroke [4]. In the past clinical work, both patients and medical workers tend to pay more attention to the secondary prevention of non-minor stroke patients, ignoring the poor prognosis of minor stroke. According to the data of the Third China National Stroke Registry (CNSR-III), TIA and minor stroke (a National Institutes of Health Stroke Scale (NIHSS) score≤5) account for about 73% of acute ischemic stroke cases. Therefore, it was essential to predict the prognosis of patients with TIA and minor stroke, find risk factors, identify high-risk patients, and accurately carry out early intervention for patients with TIA and minor stroke. With the improvement of computer computing power, the advent of the era of big data, and the update of algorithms, machine learning has made good progress in disease prediction. However, there were few comparative studies on multiple tree model machine learning algorithms. This study used the CNSR-III database to focus on factors related to the 90-day prognosis of TIA and minor stroke patients and compared the predictive performance of machine learning models and the Logistic model to provide references for related research and clinical work.

## Method

### Data availability statement

All anonymized data in this study could be shared by request from any qualified investigator.

### Study design and patients

The CNSR-III database was a nationwide prospective clinical registry of ischemic stroke or TIA in China based on etiology, imaging, and biological markers. The detailed study design of the CNSR-III trial has been described elsewhere [5]. Between August 2015 and March 2018, the CNSR-III recruited consecutive patients with ischemic stroke or TIA from 201 hospitals that covered 22 provinces and four municipalities in China. Written informed consent was obtained from the patients or their legal representatives. Clinical data were collected prospectively using an electronic data capture system by face-to-face interviews. Brain imaging, including brain magnetic resonance imaging (MRI) and computed tomography (CT), were completed at baseline. Blood samples were collected and biomarkers were tested at baseline. The registry recruited consecutive patients who met the following criteria: age >18 years; ischemic stroke or TIA; within 7 days from the onset of symptoms to enrolment; Acute ischemic stroke was diagnosed according to the World Health Organization (WHO) criteria [6] and confirmed by MRI or brain CT. Patients who had silent cerebral infarction with no manifestation of symptoms and signs or who refused to participate in the registry were excluded. The study was conducted in accordance with the Declaration of Helsinki (as revised in 2013). The study was approved by the ethics committee of Beijing Tiantan Hospital (No.: KY2015-001- 01) and all study centers gave ethical approval of the study protocol. Written consents were obtained from all participants or their legal representatives.

In this study, minor stroke was defined as an NIHSS score≤5. There were 15,166 patients in CNSR-III, and 4086 patients with an NIHSS score >5 were excluded.

There were 11,080 patients with TIA and minor stroke (an NIHSS score≤5). 113 patients were excluded (Including patients whose mRS score was missing for 90-day in the follow-up data). A total of 10967 patients were eligible for the study. Supplementary Figure 1 shows a detailed flow chart for the study population selection from CNSR-III.

### Baseline variables

For baseline variables, we investigated demographic characteristics (sex, age, BMI, race, family income/monthly, education level, living conditions), smoking history, drinking history (≥20g/day), physiological data (systolic blood pressure, diastolic blood pressure, heart rate), medical history (including stroke, hypertension, diabetes, heart disease and lipid metabolism disorders),secondary prevention treatment, in-hospital evaluation and education (including swallowing function, Limb rehabilitation and stroke-related education), laboratory data, neurological severity (admission and discharge), mRS score (admission and discharge) and TOAST classification. Finally, a total of 44 variables were included as baseline variables for analysis (Supplementary Table 1). Neurological severity was scaled by the National Institutes of Health Stroke Scale score.

### Clinical outcomes

The clinical outcome of this study was poor prognosis at 90-day. In this study, a poor prognosis was defined as the modified Rankin Scale (mRS) ≥ 3, and 0-2 was defined as a good prognosis

### Classification algorithm

We used various machine learning techniques to predict poor prognosis at 90-day: CatBoost (CB) [7], XGBoost (XGB) [8], Gradient Boosting Decision Tree (GBDT) [9], Random forest (RF) [10], and AdaBoost (Ada) [11].

CB: CatBoost is an innovative ordered gradient boosting algorithm, which uses ordered target-based statistics for categorical features processing and permutation strategies to avoid prediction shift. Its base learner was an oblivious tree and each tree corresponds to a partition of the feature space. The model learns the feature space partition at each training iteration and finally obtains the aggregated data as a classification result [7].

XGB: XGBoost was a Boosting library developed by Chen Tianqi of the University of Washington in 2016. It has both a linear scale solver and a tree learning algorithm. XGBoost was a second-order Taylor expansion of the loss function, and a regular term was added to the objective function to find the optimal solution as a whole, which was used to weigh the decline of the objective function and the complexity of the model, avoid overfitting, and improve the model The efficiency of the solution [8].

GBTD: The decision tree used in GBDT was a regression tree. The goal of each training was to reduce the error of the last training and finally get the minimum error. The model uses the gradient descent method to reduce the error [9, 12].

RF: RF was an integrated supervised learning method, which consists of multiple decision trees corresponding to different sub-data sets. Calculate the results for each tree, and get the average of the predicted results. This approach allows reducing variance in decision trees [10, 13].

Ada: The basic idea of the AdaBoost algorithm was to classify a group of weak learners through weighted majority voting (or sum). It takes into account the mistakes of previous weak learners and repeatedly updates the data [11, 14, 15].

### Data preprocessing

Missing value processing: Continuous variables were filled using linear imputation, and categorical variables were filled using mode. Most of the 44 variables had no missing values, and 4 variables had missing values greater than 5%. We examined the distribution of the laboratory data after imputation of the variables used in the modeling and found no significant difference in the distribution of the data before and after imputation (Supplementary Tables 1 and 3).

### Feature selection

Logistic regression was used for feature selection in our study. Logistic regression was the most commonly used model for characterizing the relationship between a dependent variable and one or more explanatory variable. LR models had a long and well-known theoretical and computational background, and their regression parameters and language were generally accepted. In the training set, we used univariate logistic analysis to compare baseline characteristics of 90-day prognosis. The risk factors selected by univariate analysis were included in multivariate analysis using stepwise regression method. Variables with *P* < 0.05 were used as predictors for the establishment of the 90-day poor prognosis prediction model in this study.

### Statistical analysis

Continuous variables were expressed as the mean ± standard deviation, and classification variables were expressed as a percentage. After data preprocessing, the good prognosis group (mRS≤2) and the poor prognosis group (mRS≥3) were randomly grouped according to a ratio of 70:30, divided into a training set and test set, repeated five times. The test set was only used for model testing. We use GridSearch CV for 5-fold cross-validation tuning in the training set. After tuning each model to the optimum in the training set, we performed model evaluation on a clean test set to test the predictive performance of the five models established in this study. Supplementary Table 2 showed the parameters involved in each model in this study. Data analysis application SAS software (SAS9.4) and Python software (Python v3.6.8) completed. When comparing the predictive performance of different models in this study, the comparison and evaluation were mainly conducted from the two aspects of discrimination and calibration. In this study, the discriminative index was the area under the curve (AUC). The higher the AUC value, the higher the discriminative degree of the model. The calibration index of this study adopts Brier score [16] (scoring range was 0 ~ 1). The closer the Brier score was to 0, the better the calibration of the model. Two-sided probability values <0.05 were considered statistically significant.

## Result

### Demographic and clinical characteristics

A total of 15166 patients were registered to the cohort during the study period. After excluding 4086 patients with NIHSS score >5 and 113 patients with missing clinical outcomes(90-day mRS), 10967 patients were finally included (Supplementary Figure 1).

The mean age of the 10967 patients was 61.77±11.18 years and 30.68% were female (Supplementary Figure 1 and Table 1). The training set included 7676 patients and the test set included 3291 patients. We performed feature selection in the training set (*n*=7676). First, we performed univariate analysis in the training set and found that 28 variables (*P*<0.05) differed between patients with and without a poor functional outcome at 90-day (based on 44 baseline variables). They were demographics (including sex, age, family income/monthly and education level), smoking history, drinking history (≥20g/day), physiological data (systolic blood pressure and heart rate),medical history (including stroke, hypertension, diabetes and heart disease), secondary prevention treatment, in-hospital evaluation (swallowing function, Limb rehabilitation), laboratory data, neurological severity (admission and discharge), mRS score (admission and discharge) and TOAST classification. Table 1 showed the total population and baseline data for both groups.

**Table 1:** Baseline data for the total population and for both groups

	Total *N*=10967	mRS(0-2) *N*=10234(93.32)	mRS(3-6) *N*=733(6.68)	*P* value
Demographics				
Female, n (%)	3365(30.68)	3077(30.07)	288(39.29)	<0.0001
Age, mean (SD)	61.77±11.18	61.38±11.03	67.21±11 .79	<0.0001
Family income/monthly, n (%)				0.0108
<700 Yuan	565(5.15)	525(5.13)	40(5.46)	
700~1500Yuan	1511(13.78)	1424(13.91)	87(11.87)	
1501~2300Yuan	2344(21.37)	2181(21.31)	163(22.24)	
>2300Yuan	3894(35.51)	3657(35.73)	237(32.33)	
Unknown	2653(24.19)	2447(23.91)	206(28.10)	
Education level, n (%)				0.0264
college or above	1021(9.31)	967(9.45)	54(7.37)	
high school	2242(20.44)	2100(20.52)	142(19.37)	
junior school	3229(29.44)	3061(29.91)	168(22.92)	
primary school	2175(19.83)	2017(19.71)	158(21.56)	
illiteracy	730(6.66)	643(6.28)	87(11.87)	
Unknown	1570(14.32)	1446(14.13)	124(16.92)	
smoking history, n (%)	3509(32.00)	3346(32.69)	163(22.24)	<0.0001
Drinking history(≥20g/day), n (%)	1558(14.21)	1485(14.51)	73(9.96)	0.0007
Physiological data, mean (SD)				
Systolic blood pressure	149.67±21.87	149.43±21.75	153.00±23.22	<0.0001
Heart rate	75.24±11.19	75.12±11.16	76.91±11.49	<0.0001
medical history, n (%)				
Stroke	2317(21.13)	2085(20.37)	232(31.65)	<0.0001
Hypertension	6865(62.60)	6366(62.20)	499(68.08)	0.0015
Diabetes	2532(23.09)	2323(22.70)	209(28.51)	0.0003
Heart disease	1383(12.61)	1267(12.38)	116(15.83)	0.0067
secondary prevention treatment, n (%)				
Anti-platelet	10637 (97.61)	9938(97.73)	699(95.88)	0.0019
Anticoagulation	905(8.30)	805(7.92)	100(13.72)	<0.0001
Antidiabetic	2721(24.97)	4767(46.88)	355(48.70)	0.0014
Expansion treatment	1526(14.01)	1400(13.77)	126(17.28)	0.0081
swallowing function, n (%)	273(2.84)	208(2.32)	65(9.73)	<0.0001
Limb rehabilitation, n (%)	7466(68.08)	6913(67.55)	553(75.44)	<0.0001
Laboratory data, mean (SD)				
FBG	6.34±2.53	6.31±2.51	6.73±2.76	0.0002
Creatinine	73.01±29.79	72.80±28.57	75.91±43.27	0.0091
D-dimer	1.39±2.37	1.36±2.29	1.89±3.24	<0.0001
C-reactive protein	5.84±21.96	5.47±21.26	10.99±29.49	<0.0001
Triglycerides	1.69±2.83	1.70±2.91	1.655±1.09	0.0069
Neurological severity				
admission NIHSS score, median (IQR)	2(1-4)	2(0-4)	3(2-4)	<0.0001
Discharge NIHSS score, mean (IQR)	1(0-2)	1(0-2)	3(1-6)	<0.0001
Admission mRS, mean (IQR)	1(1-2)	1(1-2)	2(1-3)	<0.0001
Discharge mRS, mean (IQR)	1(0-1)	1(0-1)	3(1-4)	<0.0001
TOAST classification, n (%)				<0.0001
LAA, n (%)	2509(22.88)	2268(22.16)	241(32.88)	
CE, n (%)	573(5.22)	520(5.08)	53(7.23)	
SAO, n (%)	2561(23.35)	2458(24.02)	103(14.05)	
ODC, n (%)	128(1.17)	117(1.14)	11(1.50)	
UND, n (%)	5196(47.38)	4871(47.60)	325(44.34)	

*Abbreviations*: *NIHSS* National Institutes of Health Stroke Scale, *FBG* Fasting blood glucose, *TOAST* The Trial of Org 10172 in Acute Stroke Treatment (TOAST) criteria, *LAA* Large-artery atherosclerosis, *CE* Cardioembolism, *SAO* Small-vessel occlusion, *ODC* Stroke of other determined etiology; and UND:stroke of undermined etiology [17], *IQR* Interquartile range, *SD* Standard deviation

### Determining predictors of poor prognostic outcome

In the training set, the risk factors selected by the univariate analysis were included in the multivariate analysis. Multivariate Logistic regression results showed that sex, age, stroke history, heart rate, D-dimer, creatinine, TOAST classification, admission mRS, discharge mRS and discharge NIHSS score were related to the 90-day poor prognosis (*P*<0.05). The above factors can be used as predictive indicators to build a predictive model. In TOAST classification, LAA was set as a control variable. In laboratory indicators, D-dimer and creatinine were risk factors for a poor prognosis of 90-day (OR=1.051, OR=1.003, respectively). Compared with males, females were risk factors for a poor prognosis of 90-day (OR= 1.226),other factors were showed in Table 2 below.

**Table 2:** Association between predictors and poor functional outcome in multivariable analysis (In the training set)

	*β*	OR	*P*	95%CI
Sex,female	0.2034	1.226	0.0296	1.020~1.472
AGE	0.0405	1.041	<0.0001	1.033~1.050
Stroke history	0.3114	1.365	0.0011	1.132~1.647
Heart rate	0.0156	1.016	<0.0001	1.008~1.023
D-dimer (μg/ml)	0.0496	1.051	<0.0001	1.026~1.076
Creatinine (μmol/L)	0.00349	1.003	0.0045	1.001~1.006
TOAST classification	-	-	<0.0001	
LAA	-	-		
CE	-0.2002	0.819	0.2868	0.566~1.183
SAO	-0.6574	0.518	<0.0001	0.398~0.674
ODC	-0.0435	0.957	0.9080	0.458~2.001
UND	-0.2310	0.794	0.0244	0.649~0.971
Admission mRS	0.1471	1.159	<0.0001	1.074~1.250
Discharge mRS	0.8289	2.291	<0.0001	2.077~2.527
Discharge NIHSS score	0.1495	1.161	<0.0001	1.114~1.210

*Abbreviations*: *OR* Odds ratio, *NIHSS* National Institutes of Health Stroke Scale, *TOAST* The Trial of Org 10172 in Acute Stroke Treatment (TOAST) criteria, *LAA* Large-artery atherosclerosis, *CE* Cardioembolism, *SAO* Small-vessel occlusion, *ODC* Stroke of other determined etiology; and UND Stroke of undermined etiology [17], *IQR* Interquartile range, *SD* Standard deviation

### Predictive performance of different models

**Fig. 1:**
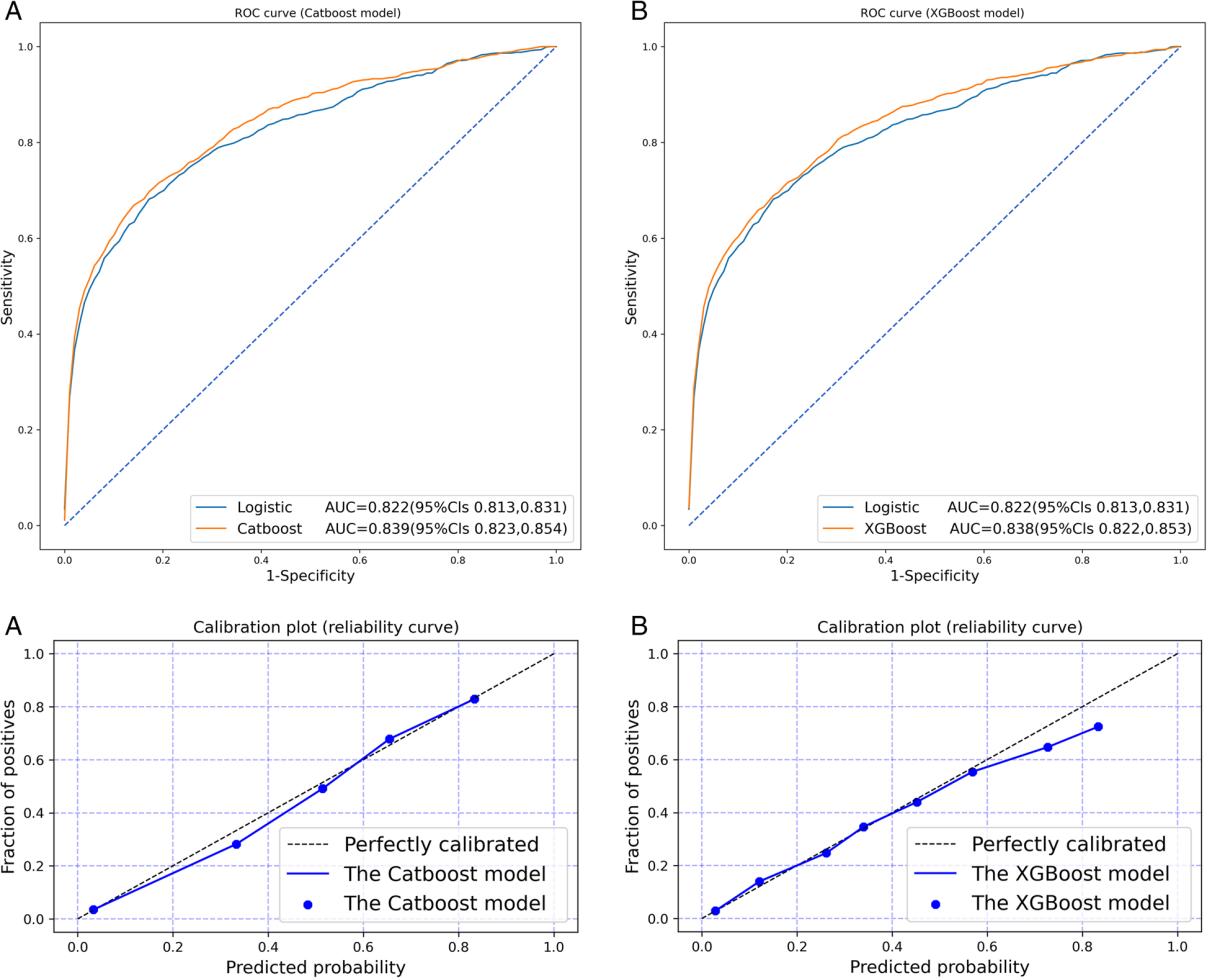
Calibration plots for prediction of stroke outcome at 90-day on test sets: **A** the Catboost model, **B** the XGBoost model

**Fig. 2:**
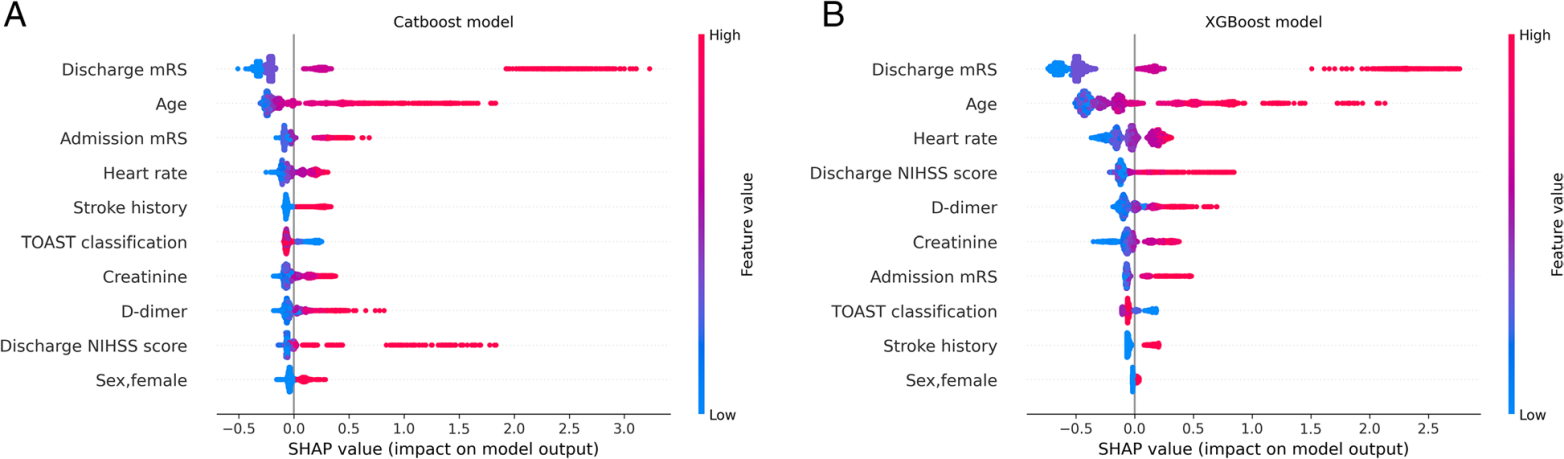
SHapley Additive exPlanations (SHAP) plots, ranking plot of shap values on test sets. The blue to red color represents the feature value (red high, blue low). The x-axis measures the impacts on the model output (right positive, left negative). (**A**) the Catboost model, (**B**) the XGBoost model

In the training set, We used the multivariate Logistic regression model as a feature selection method, and determined 10 variables as predictors to establish different prediction models. Five tree model classifiers, namely CB, XGB, GBDT, RF and Ada, were trained on the training data set repeated 5 times, and 5-fold cross-validation was performed to adjust the optimal parameters. Finally, the five optimal models adjusted training sets will be evaluated in test sets. The results of AUC, accuracy, PPV, NPV, F1-score, and Brier score in the test set were showed in Table 3. Compared with other machine learning classifiers, and the CB model had the highest AUC of 0.839, followed by XGB, GBDT, RF, and Ada models (0.838, 0, 835, 0.832, 0.823, respectively). The AUC of all machine learning classifiers was higher than the Logistic model (AUC=0.822). Supplementary Figure 2 showed the ROC curve and the area under the curve (AUC) of each machine learning classifier in test sets compared with the Logistic model. The prediction performance of the CB and XGB model was better than the Logistic model, and the difference was statistically significant (*P*<0.05). Figure 1 showed ROC curve and calibration plots of CB and XGB models on test sets. In terms of calibration, CB, XGB, and GBDT had the best calibration (Brier scores were all 0.047), and the Ada model had the worst calibration (Brier score=0.159) (Table 3). In addition, Supplementary Figure 3 showed calibration plots of each model on test sets. SHAP values for the two models (the CB model and the XGB model) were assessed in the test set, and are shown in Fig. 2, respectively.

**Table 3:** Test sets result of machine learning models and the Logistic model on 90-day stroke outcome prediction

Model	Auc(95%CI)	Accuracy(95%CI)	PPV(95%CI)	NPV(95%CI)	F1-score(95%CI)	Brier score(95%CI)
CB	0.839(0.823,0.854)	0.942(0.938,0.947)	0.660(0.605, 0.716)	0.951(0.948,0.954)	0.404(0.382,0.427)	0.047(0.044,0.050)
XGB	0.838(0.822,0.853)	0.943(0.939,0.947)	0.664(0.595,0.734)	0.952(0.949,0.955)	0.423(0.394,0.452)	0.047(0.044,0.050)
GBDT	0.835(0.820,0.850)	0.942(0.938,0.946)	0.648(0.589,0.707)	0.951(0.948,0.954)	0.403(0.377,0.428)	0.047(0.044,0.050)
RF	0.832(0.815,0.849)	0.940(0.937,0.943)	0.659(0.595,0.723)	0.946(0.944,0.949)	0.326(0.303,0.348)	0.048(0.045,0.051)
Ada	0.823(0.810,0.837)	0.941(0.938,0.945)	0.636(0.570,0.702)	0.951(0.949,0.953)	0.395(0.366,0.424)	0.159(0.157,0.161)
LR^a^	0.822(0.813,0.831)	0.941(0.938,0.945)	0.685(0.635,0.735)	0.947(0.944,0.951)	0.348(0.320,0.376)	0.048(0.046,0.051)

^a^*LR* Logistic regression model

## Discussion

Machine learning (ML) methods have gained increasing popularity in medical research. Machine learning-based algorithms may be used for screening, diagnostic, or prognostic purposes. ML methods have been tested in several medical conditions to predict a future health state in cardiovascular medicine. This study aimed two-fold: On the one hand, the training set of this study was used to discover the related factors of poor 90-day prognosis in patients with TIA and minor stroke, which provided reference for related research and clinical work. On the other hand, factors found in training sets were used as indicators for predicting poor patient prognosis at 90-day. 90-day poor prognosis prediction models for TIA and minor stroke patients were constructed, and the prediction performance of the ML algorithm and the Logistic model was compared.

There were two significant findings of this study:

1. We performed univariate and multivariate analyses of patients with TIA and minor stroke in the training set and found that the 90-day prognosis prediction of patients with TIA and minor stroke was determined by many factors: sex, age, stroke history, heart rate, D-dimer, creatinine, TOAST classification, admission mRS, discharge mRS, and discharge NIHSS score.
2. The discrimination and the calibration of each model were good. The discrimination (AUC) between the CB model and the XGB model was better than the Logistic model (*P*<0.05), and CB model has the best prediction performance.

In previous studies, the prognosis of stroke was affected by factors such as stroke severity (NIHSS score) [18, 19], age [18–21], sex [20–22], and comorbidities [22–24]. In this study, the 90-day prognosis of TIA and minor stroke patients were determined by many factors. Such as sex, age, stroke history, heart rate, D-dimer, creatinine, TOAST classification, admission MRS, discharge MRS , and discharge NIHSS score.

In this study, we found that the risk of 90-day poor prognosis was higher for older TIA and minor patients, and the risk of 90-day poor prognosis was higher for females than for males. The human body's natural aging process was irreversible with age Therefore, age and sex were non-intervention factors. In addition, the proportion of stroke history in the poor prognosis group was significantly higher than that in the good prognosis group, suggesting that stroke history was a predictor of the 90-day poor prognosis. Previous studies had shown that high heart rate was an independent risk factor for stroke and cardiovascular and cerebrovascular events. Patients with high a heart rate had a significantly higher incidence of stroke and adverse cardiovascular and cerebrovascular outcomes [25–28]. This study found that TIA and minor patients with high heart rate levels had a higher risk of poor prognosis at 90-day. This might be related to the high level of heart rate that increased myocardial oxygen consumption and reduces cardiac reserve. It was also possible that the excessive activation of sympathetic nerves damages the cardiovascular system through multiple mechanisms, leading to a poor prognosis of stroke patients at 90-day. This study suggested that the heart rate level was related to the 90-day poor prognosis prediction for patients with TIA and minor stroke.

This study found that a high level of creatinine at admission was associated with a poor prognosis at 90-day, and patients with a high creatinine level were at higher risk of a poor prognosis at 90-day. In recent years, studies found a specific correlation between creatinine and nerve damage, but there were few clinical correlations between creatinine and nerve damage and stroke prognosis [29]. Unlike previous studies [30], this study suggested that early detection of creatinine was of great significance for the prognosis prediction of patients with TIA or minor stroke. Although the D-dimer levels were elevated in patients with ischemic stroke, the relationship with a poor prognosis was unclear. Elevated D-dimer levels may be related to several factors [31]. (1) The level of D-dimer in stroke patients was positively correlated with the infarct volume; (2) The high level of D-dimer was an indicator of systemic hypercoagulability. (3)D-dimer could activate inflammation. (4) The high level of D-dimer could indicate that the thrombus has a higher tolerance for endogenous fibrinolytic system thrombolysis. (5) D-dimer levels were high, often accompanied by venous thrombosis of the lower extremities. However, D-dimer had the advantages of high in vitro activation tolerance and long half-life and rarely increased in the blood-brain barrier of healthy people [32]. This study suggested that high levels of D-dimer were associated with a poor prognosis at 90-day. Patients with high D-dimer levels were at high risk of poor prognosis at 90-day. Previous studies had also showed that higher D-dimer levels could predict the prognosis of acute myocardial infarction [33]. In short, the early detection of changes in patients' creatinine and D-dimer levels has important application value in predicting the outcome and prognosis of TIA and minor stroke patients. At present, studies had showed that TOAST classification was related to the severity and prognosis of stroke patients [34]. This study suggested that TOAST classification was correlated with the prognosis prediction of TIA or minor stroke patients. Therefore, early determination of the TOAST classification of TIA and minor stroke patients was of great significance for secondary prevention and predicting the poor prognosis of patients. NIHSS score was currently the most commonly used scale for acute ischemic stroke in the world. The NIHSS score was closely related to the patient's cerebral infarction volume, location, and other factors. It could be used to efficiently and effectively evaluate the degree of neurological impairment in patients with acute ischemic stroke. Patients discharged from the hospital with a high NIHSS score were generally more severely ill and had a larger brain tissue infarction volume, which had a lot to do with the poor prognosis of patients [35]. In addition, this study showed that the mRS score at admission and discharge was related to the 90-day prognosis of patients with TIA and minor stroke, and the higher the score, the higher the risk of adverse prognosis.

All six models showed good discrimination in this study (AUC>0.80), which was consistent with previous studies [36–39]. Among them, the CB model had the highest prediction performance, followed by XGB, GBDT, RF, Ada, and Logistic (AUC were 0.839, 0.838, 0.835, 0.832, 0.823, and 0.822, respectively), where CB and XGB were better than the Logistic model, the difference was statistically significant (*P*<0.05). Conventionally, AUC values>0.70 were considered to represent moderate discrimination, values>0.80 good discrimination, and values>0.90 excellent discrimination. In terms of calibration, the Brier score of each model was better, and the CB, XGB and GBDT models have the best correction effect (Brier score=0.047). In addition, Supplementary Figure 3 showed calibration plots of each model on test sets. It can be seen that the calibration curves of each model were better, especially the CB model and the XGB model, indicating that there was no overfitting on test sets.

In previous studies, when comparing ML models and the traditional Logistic model, ML models were superior to logistic model likely due to the features instead of the algorithm itself [36, 40–42], because different models often use different predictors. In addition to the predictors, the characteristics of the algorithm are also important factors to consider when selecting a model suitable for medical application. Unlike most of previous studies in machine learning, this study used the same features selected by the logistic model. Our finding at ML models outperformed logistic model in that circumstance. In recent years, many studies had used SHAP value to explain the "black boxes", and its core idea is to calculate the marginal contribution of features to the model output. Although the degree of contribution and the impact on outcome variables can be seen in the SHAP plot, in clinical studies, there is a lack of quantitative explanations for the specific impact of each clinical variable. Logistic regression, as a traditional statistical model, has the advantage of being able to explain the relationship between variables and outcomes well compared to pure machine learning algorithms for feature selection. In the process of feature selection, traditional logistic regression can use its confidence interval and OR indicator to specifically represent the relationship between predictors and outcomes, making up for the problem of machine learning “black boxes”.

This study showed that compared with the Logistic model, the CB model showed the highest AUC. This result showed that the CatBoost algorithm can well predict patients with poor 90-day prognosis from TIA and minor stroke patients. CatBoost was a new integrated algorithm based on decision tree gradient lifting developed by researchers and engineers of Yandex in Russia. Previously, the two mainstream algorithms in the Boosting family were XGBoost and LightGBM, and according to official evaluation, the new CatBoost member model in the Boosting family had better performance than the above two algorithms. It was also consistent with the results of this study. CatBoost’s success might have been explained by its ability to process categorical features and model feature combinations. Additionally, CatBoost’s new capacity in undertaking feature combinations increased its nonlinear modeling abilities. In addition, although the LR algorithm performed well, it may also be because we used the stepwise regression method to screen the characteristic variables. Perhaps the CatBoost algorithm will be more convenient when there are more feature variables, a larger amount of data, or even multivariate heterogeneous data, and the performance difference between the two algorithms will be greater. As an emerging algorithm, CatBoost has unique advantages. It competes with any leading machine learning algorithm and handles categorical features automatically, requiring less hyperparameter tuning, enhancing model stability and reducing the possibility of overfitting.

As our analysis showed, machine learning models, especially CatBoost algorithm, showed promising results in predicting poor outcome in patients with 90-day TIA and minor stroke. Due to the particularity of clinical research, it is extremely important to select variables based on prior knowledge, and to use stepwise regression with more interpretability for features selection. Although our study used only 10 predictors for modeling, the machine learning model, especially the CB model, showed better predictive performance without sacrificing accuracy. And due to the low cost of the number of features, it is more convenient to perform external verification and input into Clinical Decision Support System (CDSS) for clinical practical application prediction in the future. The knowledge-driven model of combining new algorithms in machine learning may complement the purely data-driven approach of previous research in the field of machine learning for disease. I believe that our research can be applied to electronic health records to provide services for doctors, health care workers.

This study has several limitations. First, although the ML algorithm could have high accuracy and AUC performance, especially CB models, we still need more external validation to check the robustness of ML model. Second, it could be combined with a better selection algorithm to improve accurate prediction In the future. Third, imaging and omics data were not included in this study, which may limit the predictive performance to some extent. Fourth, this study performed a single imputation of missing values, which will inevitably lead to certain bias, but if the missing values are deleted, certain selection bias cannot be avoided; Since mehtods of directly testing missing at random is not available yet, we are not confident to state that variables with >5% missing values are missing at random [43]. Nevertheless, we believe that the selection bias would be minimized in this study as the baseline characteristics between included and excluded patients were largely comparable.

We will further explore the adaptation conditions of different models for ischemic stroke and conduct comprehensive studies on the development of predictive models and predictive performance to provide a more comprehensive reference for establishing a perfect prognosis prediction of ischemic stroke.

## Supplementary Information

**Additional file 1.**

**Additional file 2.**

**Additional file 3.**

**Additional file 4.**

**Additional file 5.**

**Additional file 6.**

## Data Availability

The datasets used and/or analysed during the current study are available from the corresponding author on reasonable request.
