# Supplementary figures and images for: Machine learning is an effective method to predict the 90-day prognosis of patients with transient ischemic attack and minor stroke
Source: BMC Med Res Methodol. 2022 Jul 16;22:195. doi: 10.1186/s12874-022-01672-z (PMC9287991; doi:10.1186/s12874-022-01672-z)

Data Supplement


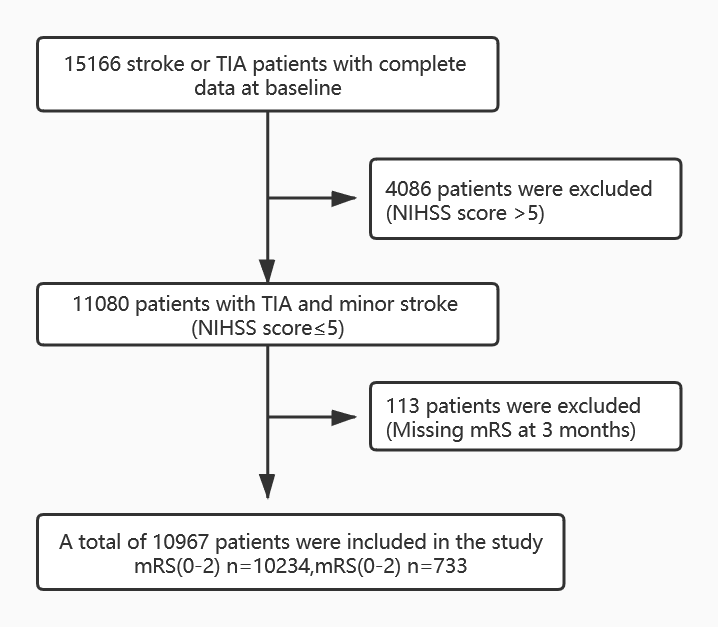


Supplementary Figure 1.Schematic illustration of the study design.

Supplement: Supplementary file 1 — Additional file 1. [file 12874_2022_1672_MOESM1_ESM.docx]
