# Supplementary material for: Machine learning is an effective method to predict the 90-day prognosis of patients with transient ischemic attack and minor stroke
Source: BMC Med Res Methodol. 2022 Jul 16;22:195. doi: 10.1186/s12874-022-01672-z (PMC9287991; doi:10.1186/s12874-022-01672-z)
Supplement: Supplementary file 2 — Additional file 2. [file 12874_2022_1672_MOESM2_ESM.docx]

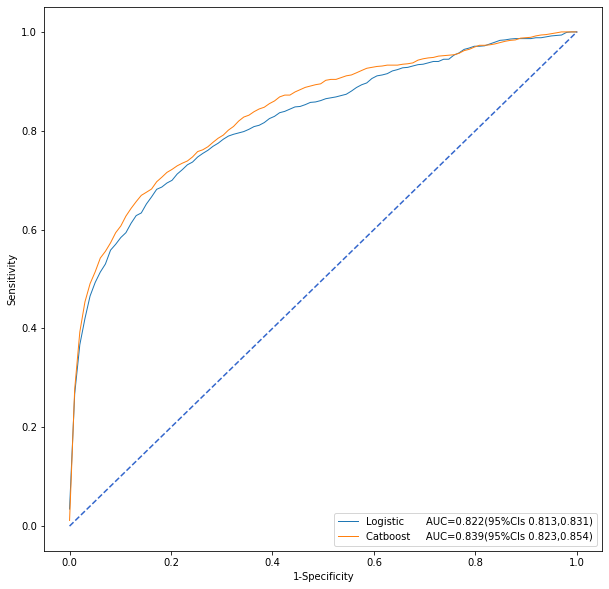

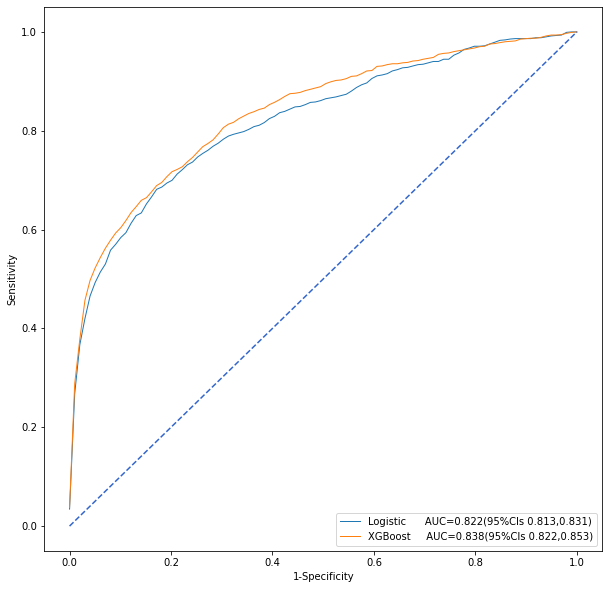

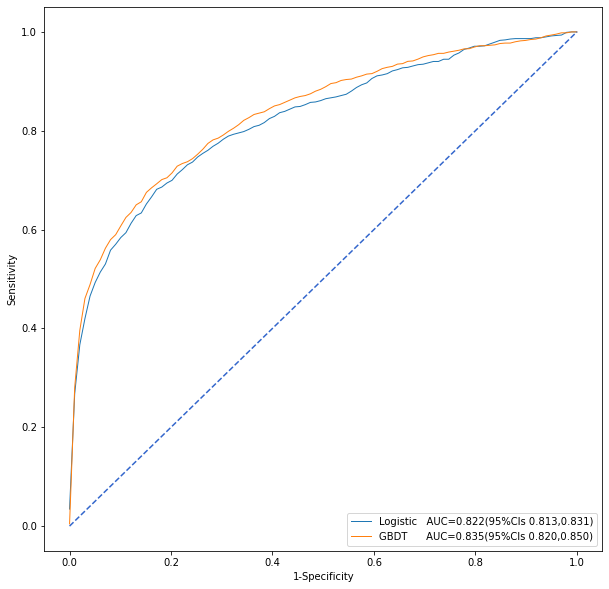

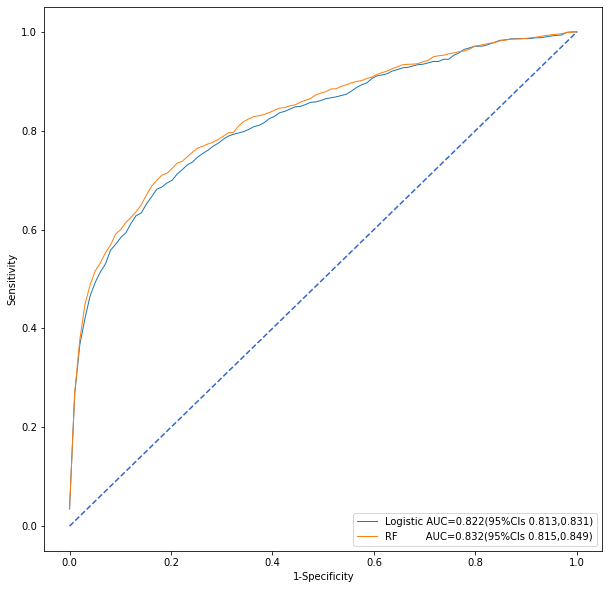

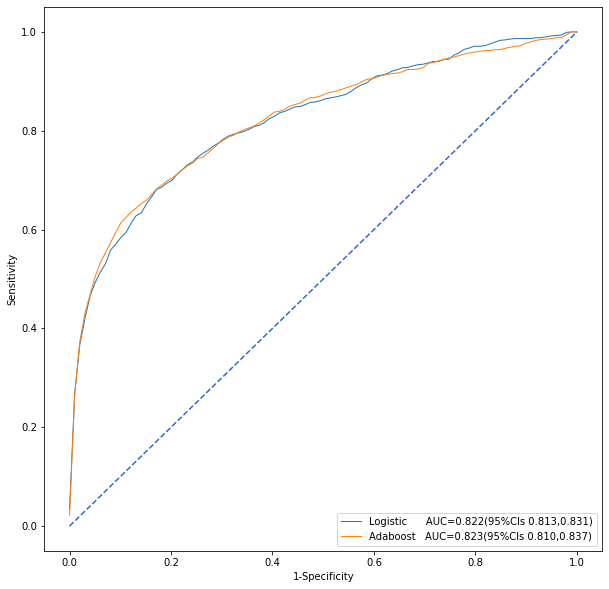


**Supplementary Figure 2.The receiver operating curve of 90-day stroke outcome prediction models on test sets; RF—random forests.**
