# Supplementary material for: Machine learning is an effective method to predict the 90-day prognosis of patients with transient ischemic attack and minor stroke
Source: BMC Med Res Methodol. 2022 Jul 16;22:195. doi: 10.1186/s12874-022-01672-z (PMC9287991; doi:10.1186/s12874-022-01672-z)
Supplement: Supplementary file 3 — Additional file 3. [file 12874_2022_1672_MOESM3_ESM.docx]

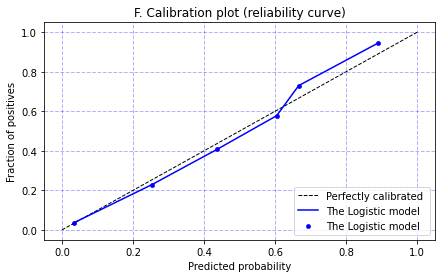

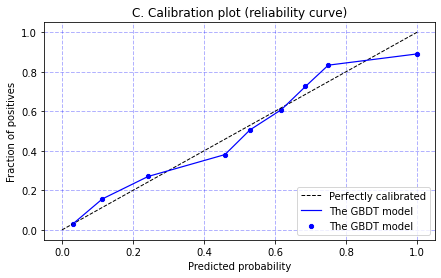

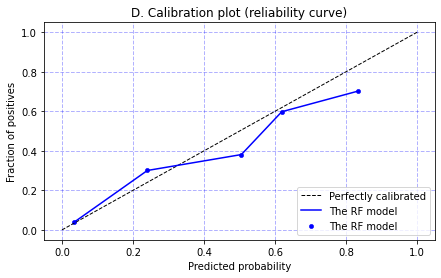

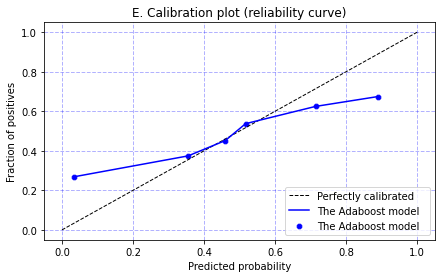

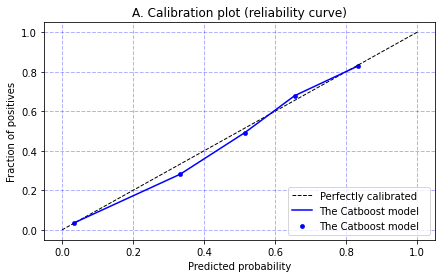

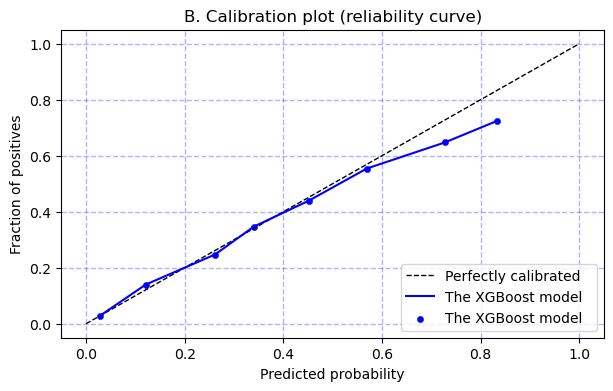


**Supplementary Figure 3.**

**Calibration plots for prediction of TIA and minor stroke outcome at 90-day on test sets: A the Catboost model, B the XGBoost model,C the GBDT model, D Random Forest model, E the Adaboost model, F the Logistic regression model.**
