# Supplementary material for: Machine learning is an effective method to predict the 90-day prognosis of patients with transient ischemic attack and minor stroke
Source: BMC Med Res Methodol. 2022 Jul 16;22:195. doi: 10.1186/s12874-022-01672-z (PMC9287991; doi:10.1186/s12874-022-01672-z)
Supplement: Supplementary file 4 — Additional file 4. [file 12874_2022_1672_MOESM4_ESM.doc]

**Supplementary Table 1. Baseline data of the patients**

| Baseline characteristics | Data type | Value | Missing, n (%) |
| --- | --- | --- | --- |
| Demographics |  |  |  |
| Sex,Female, n (%) | yes/no | 3365(30.7) | 0(0.00) |
| Age, mean (SD) | years | 61.8(11.2) | 0(0.00) |
| BMI,mean (SD) | kg/m2 | 24.8(3.3) | 0(0.00) |
| Race,Han, n (%) | yes/no | 10692(97.5) | 0(0.00) |
| Family income/monthly, median (IQR) | 1-4 | 4(3-4) | 0(0.00) |
| Education level, median (IQR) | 1-5 | 3(2-4) | 0(0.00) |
| Living conditions,alone,n (%) | yes/no | 536(4.9) | 0(0.00) |
| Smoking history, n (%) | yes/no | 3509(32.0) | 0(0.00) |
| Drinking history(≥20g/day), n (%) | yes/no | 1558(14.2) | 0(0.00) |
| Physiological data, mean (SD) |  |  |  |
| Systolic blood pressure | mmHg | 149.7(21.9) | 0(0.00) |
| Diastolic blood pressure | mmHg | 87.3(12.9) | 0(0.00) |
| Heart rate | min | 75.3(11.2) | 0(0.00) |
| medical history, n (%) |  |  |  |
| Stroke | yes/no | 2317(21.1) | 0(0.00) |
| Hypertension | yes/no | 6865(62.6) | 0(0.00) |
| Diabetes | yes/no | 2532(23.1) | 0(0.00) |
| Heart disease | yes/no | 1383(12.6) | 0(0.00) |
| Lipid metabolism disorders | yes/no | 906(8.3) | 0(0.00) |
| secondary prevention treatment, n (%) |  |  |  |
| rt-PA thrombolytic | yes/no | 649(5.9) | 0(0.00) |
| Urokinase thrombolysis | yes/no | 113(1.0) | 0(0.00) |
| Anti-platelet | yes/no | 10637 (97.6) | 69(0.63) |
| Anticoagulation | yes/no | 905(8.3) | 69(0.63) |
| Lipid-lowering drugs | yes/no | 10484(96.2) | 69(0.63) |
| Antioxidant | yes/no | 1820(16.7) | 69(0.63) |
| Antidiabetic | yes/no | 2721(25.0) | 69(0.63) |
| Antihypertensive | yes/no | 5122(47.0) | 69(0.63) |
| Expansion treatment | yes/no | 1526(14.0) | 71(0.65) |
| Traditional Chinese medicine | yes/no | 6461(59.3) | 70(0.64) |
| swallowing function, n (%) | yes/no | 273(2.8) | 1343(12.25) |
| Limb rehabilitation, n (%) | yes/no | 7466(68.1) | 0(0.00) |
| stroke-related education, n (%) | yes/no | 10773(98.3) | 12(0.11) |
| Laboratory data, mean (SD)* |  |  |  |
| FBG | mmol/L | 6.3(2.5) | 2074(18.91) |
| Total cholesterol | mmol/L | 4.3(1.2) | 425(3.88) |
| Creatinine | μmol/L | 73.0(29.8) | 256(2.33) |
| D-dimer | μg/ml | 1.4(2.4) | 1685(15.36) |
| HDL-C | mmol/L | 1.1(0.5) | 456(4.16) |
| C-reactive protein | mg/L | 5.9(21.9) | 2559(23.33) |
| LDL-C | mmol/L | 2.5(1.0) | 455(4.15) |
| Triglycerides | mmol/L | 1.7(2.8) | 455(4.15) |
| Uric acid | μmol/L | 309.7(89.8) | 502(4.58) |
| Neurological severity |  |  |  |
| admission NIHSS score, median (IQR) | 0-5 | 2(1-4) | 0(0.00) |
| Discharge NIHSS score, median (IQR) | 0-40 | 1(0-2) | 12(0.11) |
| Admission mRS,median (IQR) | 0-5 | 1(1-2) | 0(0.00) |
| Discharge mRS,median (IQR) | 0-5 | 1(0-1) | 12(0.11) |
| TOAST classification,median (IQR) | 1-5 | 3(2-5) | 0(0.00) |
| LAA, n (%) | 1 | 2509(22.9) | 0(0.00) |
| CE, n (%) | 2 | 573(5.2) | 0(0.00) |
| SAO, n (%) | 3 | 2561(23.4) | 0(0.00) |
| ODC, n (%) | 4 | 128(1.2) | 0(0.00) |
| UND, n (%) | 5 | 5196(47.4) | 0(0.00) |

Abbreviations: BM, body mass index; NIHSS, National Institutes of Health Stroke Scale; FBG, fasting blood glucose; TOAST, the Trial of Org 10172 in Acute Stroke Treatment (TOAST) criteria; LAA, large-artery atherosclerosis; CE,cardioembolism;SAO,small-vessel occlusion; ODC,stroke of other determined etiology; and UND:stroke of undermined etiology^[16]^; IQR:interquartile range; SD:standard deviation.

*: There was no significant difference in data distribution before and after single imputation of missing values (Supplementary Table 3 ).
