# Supplementary material for: Machine learning is an effective method to predict the 90-day prognosis of patients with transient ischemic attack and minor stroke
Source: BMC Med Res Methodol. 2022 Jul 16;22:195. doi: 10.1186/s12874-022-01672-z (PMC9287991; doi:10.1186/s12874-022-01672-z)
Supplement: Supplementary file 5 — Additional file 5. [file 12874_2022_1672_MOESM5_ESM.docx]

**Supplementary Table 2.Hyperparameter search domains and final settings.**

| **Model** | **Description of Parameters** | **Search domain** | **Final setting** |
| --- | --- | --- | --- |
| **Catboost model** |  |  |  |
| ‘iterations’ | The maximum number of trees that can be built when solving machine learning problems. | [100,250,500,1000] | 100 |
| ‘depth’ | Depth of the tree. | [1,2,3,4,5,6,9,10] | 5 |
| ‘eval_metric’ | The metric used for overfitting detection (if enabled) and best model selection (if enabled). | ‘AUC’ | ‘AUC’ |
| ‘l2_leaf_reg’ | Coefficient at the L2 regularization term of the cost function | [1,5,9,11] | 9 |
| ‘learning_rate’ | The learning rate. | [0.01,0.1,0.3] | 0.1 |
| ‘loss_function’ | The [metric](https://catboost.ai/en/docs/concepts/loss-functions) to use in training. The specified value also determines the machine learning problem to solve. | ‘Logloss’ | ‘Logloss’ |
| ‘logging_level’ | The logging level to output to stdout. | ‘Verbose’ | ‘Verbose’ |
| **XGBoost model** |  |  |  |
| ‘learning_rate’ | Learning rate, the prediction result of each tree must be multiplied by this learning rate | [0.01,0.1,0.3] | 0.1 |
| ‘min_child_weight’ | Defines the minimum sum of weights of all observations required in a child. | [1,2,3,4,5,6,7,9] | 6 |
| ‘n_estimators’ | The number of trees used to fit can also be understood as the number of iterations | [100,300,500,1000] | 500 |
| ‘seed’ | The random number seed. | default | 0 |
| ‘colsample_bytree’ | Denotes the subsample ratio of columns for each split, in each level. | [0.5,0.6,0.7,0.8,0.9,1] | 0.9 |
| ‘max_depth’ | The maximum depth ,Used to control over-fitting as higher depth will allow model to learn relations very specific to a particular sample. | [3,5,7,9] | 3 |
| ‘gamma’ | A node is split only when the resulting split gives a positive reduction in the loss function. Gamma specifies the minimum loss reduction required to make a split. | default | 0 |
| ‘reg_alpha’ | L1 regularization term on weight (analogous to Lasso regression),The algorithm runs much faster when implemented | [10,20,30,50,70,90] | 20 |
| ‘reg_lambda’ | L2 regularization term on weights (analogous to Ridge regression) | [0.1,0.2,0.3,0.4,0.6,0.8] | 0.3 |
| ‘subsample’ | Denotes the fraction of observations to be randomly samples for each tree. | [0.2,0.4,0.6,0.8,0.9] | 0.8 |
| ‘class_weight’ | The class_weight parameter determines the weight of each classification type of the sample | ‘balanced’ | ‘balanced’ |
| **GBDT model** |  |  |  |
| ‘learning_rate’ | Learning rate shrinks the contribution of each tree by learning_rate. | [0.01,0.1,0.3] | 0.1 |
| ‘n_estimators’ | The number of boosting stages to perform. Gradient boosting is fairly robust to over-fitting so a large number usually results in better performance | [50,100,300,500] | 100 |
| ‘max_depth’ | The maximum depth of the individual regression estimators. | [1,3,5,7,9] | 3 |
| ‘min_samples_split’ | The minimum number of samples required to split an internal node. | range(100,1000,100) | 1000 |
| ‘min_samples_leaf’ | The minimum number of samples required to be at a leaf node. | [6,8,10,12,14] | 10 |
| ‘subsample’ | The fraction of samples to be used for fitting the individual base learners. | [0.2,0.4,0.6,0.8,0.9] | 0.8 |
| ‘random_state | Controls the random seed given to each Tree estimator at each boosting iteration. | 10 | 10 |
| **RF model** |  |  |  |
| ‘n_estimators’ | The number of trees in the forest. | range(100,500,100) | 200 |
| ‘max_depth’ | The maximum depth of the tree. | [1,3,5,7,9] | 3 |
| ‘max_features’ | The number of features to consider when looking for the best split | range(1,10,1) | 5 |
| ‘random_state’ | random_state is the seed used by the random number generator. | 10 | 10 |
| ‘min_samples_split’ | The minimum number of samples required to split an internal node | range(10,150,10) | 70 |
| ‘min_samples_leaf’ | The minimum number of samples required to be at a leaf node | default | 1 |
| **Adaboost model** |  |  |  |
| ‘learning_rate’ | Learning rate, which represents the rate of gradient convergence | [0.01,0.1,0.2,0.3] | 0.3 |
| ‘n_estimators’ | The number of times the base classifier is promoted (cycle) is 50 times by default. If this value is too large, the model is easy to overfit; if the value is too small, the model is easy to underfit. | range(100,500,100) | 500 |
| ‘max_depth’ | Maximum depth of decision tree | [2,3,5,7,9,10,12] | 2 |
| ‘min_samples_split’ | Minimum number of samples required for subdividing internal nodes | range(50,150,10) | 100 |
| ‘min_samples_leaf’ | Minimum number of leaf nodes | range(1,20,2) | 7 |
| ‘algorithm’ | The boosting algorithm, that is, the model promotion criterion, there are two ways: SAMME, and SAMME.R. This research uses SAMME. | ‘SAMME’ | ‘SAMME’ |
| **Logistic model** |  |  |  |
| ‘penalty’ | ‘L1’ or ‘L2’,The parameter used to prevent over-fitting, the principle is to add a regular term after the loss function. | ‘L2’ | ‘L2’ |
| ‘solver’ | Which method is used to seek the local optimum of the minimum loss function.Such as ‘liblinear’, ‘lbfgs’, ‘newton-cg’, ‘sag’, ‘sage’.This research uses 'liblinear'(Dichotomies, supporting 'L1' and 'L2') | ‘liblinear’ | ‘liblinear’ |

Note: Other parameters not listed are default parameters.
