# Supplementary material for: Machine learning is an effective method to predict the 90-day prognosis of patients with transient ischemic attack and minor stroke
Source: BMC Med Res Methodol. 2022 Jul 16;22:195. doi: 10.1186/s12874-022-01672-z (PMC9287991; doi:10.1186/s12874-022-01672-z)
Supplement: Supplementary file 6 — Additional file 6. [file 12874_2022_1672_MOESM6_ESM.docx]

**Supplementary Table 3** **Laboratory data distribution before and after imputation**

| Baseline characteristics | Missing, n (%) | Before imputation | After imputation |
| --- | --- | --- | --- |
| Laboratory data, mean (SD) |  |  |  |
| FBG | 2074(18.91) | 6.3(2.5) | 6.3(2.4) |
| Total cholesterol | 425(3.88) | 4.3(1.2) | 4.3(1.2) |
| Creatinine | 256(2.33) | 73.0(29.8) | 73.0(29.6) |
| D-dimer | 1685(15.36) | 1.4(2.4) | 1.4(2.3) |
| HDL-C | 456(4.16) | 1.1(0.5) | 1.1(0.4) |
| C-reactive protein | 2559(23.33) | 5.9(21.9) | 5.9(21.3) |
| LDL-C | 455(4.15) | 2.5(1.0) | 2.5(1.0) |
| Triglycerides | 455(4.15) | 1.7(2.8) | 1.7(2.8) |
| Uric acid | 502(4.58) | 309.7(89.8) | 309.6(88.7) |
